# Patient-Self Inflicted Lung Injury (P-SILI): An Insight into the Pathophysiology of Lung Injury and Management

**DOI:** 10.3390/jcm14051632

**Published:** 2025-02-27

**Authors:** Himanshu Deshwal, Ahmed Elkhapery, Rudra Ramanathan, Deepak Nair, Isha Singh, Ankur Sinha, Rishik Vashisht, Vikramjit Mukherjee

**Affiliations:** 1 Division of Pulmonary, Sleep and Critical Care Medicine, Department of Medicine, School of Medicine, West Virginia University, Morgantown, WV 26506, USA; 2 Department of Medicine, Rochester General Hospital, Rochester, NY 14621, USA; 3 Division of Pulmonary, Sleep and Critical Care Medicine, School of Medicine, New York University Grossman, New York, NY 10016, USA; 4 Department of Medicine, Sinai Hospital of Baltimore, Baltimore, MD 21215, USA; 5 Department of Medicine, School of Medicine, West Virginia University, Morgantown, WV 26506, USA; 6 Section of Interventional Pulmonology, Division of Pulmonary, Allergy and Critical Care Medicine, School of Medicine, Stanford University, Stanford, CA 94305, USA; 7 Division of Pulmonary and Critical Care Medicine, Macon and Joan Brock Virginia Health Sciences at Old Dominion University, Norfolk, VA 23508, USA; 8 Division of Pulmonary, Sleep and Critical Care Medicine, School of Medicine/Bellevue Hospital, New York University Grossman, New York, NY 10016, USA; vikramjit.mukherjee@nyulangone.org

**Keywords:** acute respiratory distress syndrome (ARDS), acute hypoxic respiratory failure (AHRF), patient self-inflicted lung injury (P-SILI), lung protective mechanical ventilation

## Abstract

Acute respiratory distress syndrome (ARDS) is a heterogeneous group of disease entities that are associated with acute hypoxic respiratory failure and significant morbidity and mortality. With a better understanding and phenotyping of lung injury, novel pathophysiologic mechanisms demonstrate the impact of a patient’s excessive spontaneous breathing effort on perpetuating lung injury. Patient self-inflicted lung injury (P-SILI) is a recently identified phenomenon that delves into the impact of spontaneous breathing on respiratory mechanics in patients with lung injury. While the studies are hypothesis-generating and have been demonstrated in animal and human studies, further clinical trials are needed to identify its impact on ARDS management. The purpose of this review article is to highlight the physiologic mechanisms of P-SILI, novel tools and methods to detect P-SILI, and to review the current literature on non-invasive and invasive respiratory management in patients with ARDS.

## 1. Introduction

Acute respiratory distress syndrome (ARDS) is a heterogeneous group of disease entities that are associated with acute hypoxic respiratory failure and significant morbidity and mortality. Prior to the COVID-19 pandemic, the estimated prevalence of ARDS was 10% in critically ill patients, with mortality ranging between 30 and 50% [1]. Over the past few decades, significant focus has been on phenotyping and identifying the best management strategies in ARDS, more so in mechanically ventilated patients [2]. However, once lung injury sets in, the progression of disease starts before invasive mechanical ventilation is initiated, and various important factors driving sustained lung injury are often overlooked. The concept of patient-self-inflicted lung injury (P-SILI) has garnered much interest lately. P-SILI can occur due to the pathophysiologic impact of a patient’s excessive spontaneous respiratory efforts, which can perpetuate lung injury [3]. There is an increasing understanding of the complex mechanisms leading to P-SILI. While the studies are hypothesis-generating and have been demonstrated in animal and human studies, further clinical trials are needed to identify its impact on ARDS management. Several modern clinical and investigational tools are presently being studied to identify and monitor P-SILI. The introduction of these clinical tools has dramatically changed the treatment paradigm of ARDS and allows us to individualize care for each patient.

This review highlights the concept of P-SILI, describes its complex pathophysiologic mechanism, and reviews the state-of-the-art and most recent literature on the management of ARDS and prevention of P-SILI.

## 2. Materials and Methods

A comprehensive literature review was conducted using PubMed and Medline databases, covering studies published between 2000 and 2025. The primary objective was to synthesize the current understanding of patient self-inflicted lung injury (P-SILI) within the broader context of acute respiratory distress syndrome (ARDS). The search was structured using a combination of MeSH (Medical Subject Headings) terms and free-text keywords, including “Patient self-inflicted lung injury (P-SILI)”, “acute respiratory distress syndrome (ARDS)”, “spontaneous breathing”, “ventilator-induced lung injury (VILI)”, “neuromuscular blockade (NMB)”, “driving pressure”, “positive end-expiratory pressure (PEEP)”, and “non-invasive ventilation (NIV)”. Boolean operators (AND/OR) were applied to optimize search specificity, and additional references were identified through the manual screening of bibliographies from relevant studies. The PICO (Patient/Population/Problem, Intervention, Comparison/Control, Outcome) approach was employed to formulate the aim of this narrative review: Among adult patients with or at risk for ARDS (P), what is the optimal respiratory support strategy, i.e., NIV, PEEP, NMB, driving pressure (I), compared to standard care (C), that limits barotrauma occurrence, i.e., P-SILI, VILI (O)?

The selection process followed predefined inclusion and exclusion criteria to ensure methodological rigor (Appendix A Table A1 and Table A2). Two independent reviewers screened the titles and abstracts of identified studies, with eligible full-text articles further assessed for relevance. Disagreements were resolved through discussion or by consulting a third reviewer. Extracted data included study design, patient population characteristics, intervention details, and primary and secondary outcomes related to P-SILI.

## 3. Mechanism of Patient Self-Inflicted Lung Injury (P-SILI)

**Figure 1:**
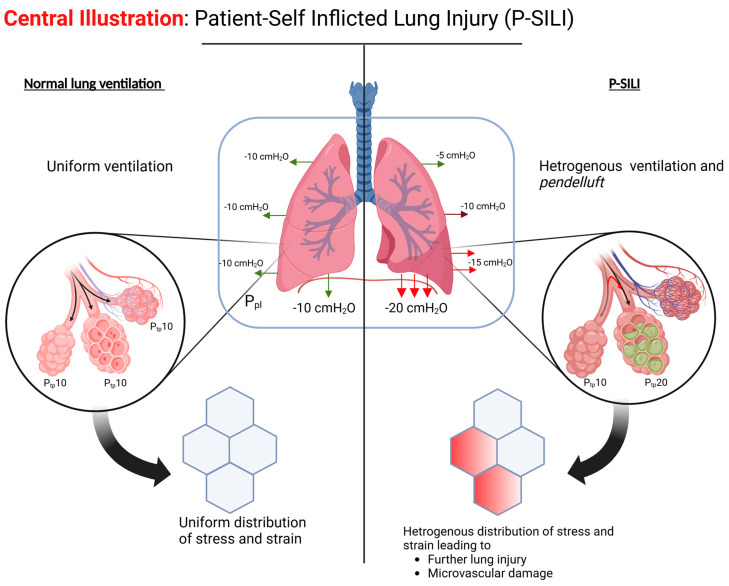
The pathophysiologic mechanism of P-SILI. (Created in BioRender. Hashem, A. (2025) https://BioRender.com/v70d189, accessed on 20 February 2025).

P-SILI occurs due to the physiologic impact of a patient’s respiratory efforts on lung injury. Five major factors play a key role in the development of P-SILI: overdistension of alveolar spaces; the impact of spontaneous breathing in lung injury; *pendelluft*; increased lung perfusion/microvascular injury; and patient–ventilator dyssynchrony (Figure 1).

### 3.1. Stress, Strain, and Overdistension

Stress is defined as the force divided by the area over which it is applied, while strain reflects the material’s response to the stress, i.e., the change in the material dimension divided by the original dimension [4]. Mechanical “stress” in the lungs is reflected by the transpulmonary pressure (PL), estimated by measuring the difference between driving pressures (plateau pressure at inspiratory hold—positive end-expiratory pressure) and pleural pressure (measured using an esophageal manometer) [5]. Strain is the change in volume compared to the initial volume [6]. Increased stress–strain beyond a certain threshold results in ventilator-induced lung damage in animal models [7].

### 3.2. Impact of Spontaneous Breathing on ARDS

Spontaneous breathing (SB) is generally encouraged over controlled mechanical ventilation in ARDS as it allows for more lung recruitment by way of active movement of the diaphragm, is associated with less hemodynamic compromise, and requires less sedation [8,9]. However, strong inspiratory effort and supraphysiologic minute ventilation can cause deleterious effects on lung function by exacerbating mechanical stress and strain [10].

SB may be beneficial in mild-to-moderate lung injury but injurious in severe lung injury. In mild-to-moderate lung injury pig models, SB is associated with higher end-expiratory lung volume (EELV) [11]. Ventilation at higher EELV causes less atelectrauma and, hence, less ventilator-associated lung injury (VILI) due to the shear stress [12]. Another study in ARDS pig models showed that SB is associated with improved aeration in the dependent lung regions, with pigs in the non-SB group having twice the amount of non-aerated lung tissue in the dependent lung regions compared to the SB group [13]. Improved aeration of the well-perfused dependent lung regions allows for better gas exchange and improved V/Q mismatch in mild–moderate ARDS.

In contrast, SB is associated with injurious levels of PL in severe ARDS, as was demonstrated by Yoshida et al. in rabbit models [14]. The use of neuromuscular blockade (NMB) curtailed the injurious effects of elevated PL in severe ARDS and achieved higher EELV, as compared to SB [15,16]. A possible mechanism of the benefit of NMB in severe ARDS is controlling the magnitude of excursion of the diaphragm and abdominal muscles, which limits the swings of PL, as demonstrated in canine models [16]. Therefore, the key to minimizing lung injury due to SB in severe ARDS is maintaining high EELV and low PL, which can be achieved by a combination of optimal PEEP setting and attenuating respiratory efforts (deep sedation/NMB).

### 3.3. Pendelluft and Regional Heterogenous Ventilation

It is important to recognize that the negative pleural pressure generated during spontaneous inspiration is not transmitted uniformly across the lung surface in injured lungs, resulting in higher PL in dependent regions compared to non-dependent areas [17]. This can result in *pendelluft* (pendulum-like movement of air), with the movement of air from non-dependent regions to dependent regions. This was demonstrated in a study by Yoshida et al., using electrical impedance tomography and dynamic computed tomography (CT) in pigs with lung injury [18]. During early inspiration, there was around 1.5–2 times greater inflation of the dependent lung regions with spontaneous breathing compared to passive breaths. This early inflation of the dependent lung regions was also accompanied by transient deflation of the non-dependent region (*pendelluft*).

Additionally, spontaneous breathing was associated with more negative intrapleural pressure in the dependent region compared to the non-dependent region. Comparable inflation of dependent lungs during paralysis required almost three-fold greater driving pressure versus spontaneous breathing. These findings were not present in non-injured lungs, suggesting that spontaneous breathing resulting in *pendelluft* may be a second-hit phenomenon resulting in worsening of acute lung injury [19].

### 3.4. Microvascular Injury Due to Transpulmonary Pressure Gradients

High negative intrapleural pressure can lead to the development of microvascular injury and negative pressure pulmonary edema [20,21,22]. This is due to positive transvascular pressure and increased movement of fluid into the lung tissue. Dreyfuss et al. showed that intermittent negative pressure ventilation (using iron lung) in a model of anesthetized male Wistar rats resulted in lung edema, similar to that seen with high-tidal-volume positive pressure ventilation [23]. A more recent study showed that spontaneous breathing was associated with more negative inspiratory pressure (Pes) and negative alveolar pressure with decreasing levels of support on pressure support ventilation (PSV) [24].

The deleterious effects of high transpulmonary pressure gradient on the pulmonary microvasculature were demonstrated through a series of experiments in rats by Katira et al., who showed that ventilation with high inspiratory pressures and no PEEP resulted in decreased right ventricular (RV) preload, increased RV afterload, and reduced RV output during inspiration, with resultant obliteration of pulmonary flow [25]. However, during expiration, there was increased RV filling and output. These large respiratory swings in the RV filling and pulmonary blood flow are thought to result in microvascular injury, evident from increased vascular permeability and resultant pulmonary edema. Ultimately, the increased RV strain resulted in the development of acute cor pulmonale and death in these experiments [25].

### 3.5. Patient–Ventilator Desynchrony

Dyssynchronous inspiratory effort during positive pressure ventilation can result in much higher transpulmonary pressures and risk of overdistention [26,27]. Sottile et al. showed that breath stacking was associated with significantly greater tidal volume compared to passive breaths [28]. These breaths are likely a result of high PL and are associated with significant stress and strain on lung parenchyma.

## 4. Novel Methods of Monitoring P-SILI

In addition to currently available clinical tools to monitor respiratory mechanics, novel techniques have been devised to monitor patient efforts and P-SILI. These include non-invasive or minimally invasive techniques, such as monitoring nasal pressure swings, airway occlusion pressure, expiratory occlusion pressure, flow index, diaphragmatic electrical activity, ventilator waveform analysis, and electrical impedance tomography (EIT). While most of these novel tools are investigational and require further validation for clinical use, they can be of importance in providing individualized care to patients with ARDS.

### 4.1. Esophageal Manometry

Esophageal manometry can help detect strong patient efforts and P-SILI by measuring esophageal pressure (Pes) as an estimate of pleural pressure. This allows for the measurement of PL, the assessment of partitioned respiratory mechanics, and the quantification of lung stress, which can guide ventilator management [18,29,30].

To detect P-SILI, dynamic changes in PL (ΔPL,dyn) can be used to estimate dynamic lung stress. This requires the real-time display of the PL waveform, with ΔPL,dyn calculated as the difference between peak and end-expiratory PL. While static ΔPL measurements can be challenging to obtain in actively breathing patients, ΔPL,dyn can provide valuable insights into lung stress. The plateau phase of PL,dyn may reflect stress on the non-dependent lung, while ΔPL,dyn likely represents the maximum dependent lung stretch. Although upper safe limits for ΔPL,dyn are uncertain, values below 15–20 cmH_2_O have been proposed, with the understanding that these limits may vary depending on lung injury severity and systemic inflammation [29].

Despite its benefits, routine bedside monitoring of esophageal pressure (Pes) is hindered by its invasive nature and potential complications, such as misplacement of the probe, technical issues, and the risk of esophageal pressure ulcers. In addition, ARDS is a heterogeneous disease, and partitioned manometric values can be misleading. Due to these limitations, alternative methods for assessing inspiratory muscle pressure (Pmus) have been developed and validated in experimental and clinical studies. These include non-invasive or minimally invasive techniques, such as monitoring nasal pressure swings, airway occlusion pressure, expiratory occlusion pressure, flow index, diaphragmatic electrical activity, ventilator waveform analysis, and electrical impedance tomography (EIT). These tools offer a simpler and more practical approach for daily clinical use but are currently investigational [29,30].

### 4.2. Electrical Impedance Tomography (EIT)

EIT is a non-invasive imaging technique that uses small alternating currents to generate functional lung images with high temporal resolution. It involves attaching electrodes around the patient’s chest, applying currents, and measuring voltage differences to estimate internal conductivity. EIT enables clinicians to visualize regional lung function, including overdistension and collapse, and optimize ventilator settings in real-time [31]. Yoshida and colleagues’ research using EIT revealed the phenomenon of *pendelluft*. Notably, *pendelluft* occurs without changes in global tidal volume or esophageal plateau pressure, making it challenging to detect during routine ventilator management [18,32,33].

### 4.3. Diaphragm Electrical Activity

Diaphragm electrical activity (EAdi) measurement offers valuable insights into respiratory drive and effort by capturing the electrical field produced by motor neurons in the crural diaphragm. This technique employs a specialized nasogastric catheter with multiple electrodes positioned at the diaphragm level, connected to dedicated ventilator software [34]. While EAdi-derived measurements show potential, they come with certain constraints when evaluating respiratory effort. These parameters are more closely linked to neural drive rather than providing a direct assessment of breathing exertion. One significant limitation is the inability of EAdi to detect the engagement of accessory muscles, which becomes particularly relevant during high-intensity breathing. This shortcoming makes EAdi less effective in gauging respiratory effort under strenuous conditions. Moreover, the absence of established reference values for EAdi-derived parameters presents a challenge in interpreting the results across different patient populations. It is not routinely utilized due to its complexity in placement and its requirement of special training. These factors collectively underscore the need for cautious interpretation and further research to enhance the clinical utility of EAdi-based assessments in respiratory monitoring [35].

### 4.4. Airway Occlusion Pressure (P0.1)

The airway occlusion pressure at 100 milliseconds (P0.1) has emerged as a valuable, non-invasive tool for assessing respiratory drive in intubated patients. This measurement represents the negative pressure generated by inspiratory muscles during the initial 0.1 s of inspiration against a blocked airway, providing insight into respiratory effort independent of muscle weakness or flow resistance [30,36]. Ventilator-displayed P0.1 measurements have shown significant correlations with other respiratory drive indicators, such as the diaphragm’s electrical activity, increased rate, and inspiratory effort as measured by esophageal pressure–time product. This parameter has demonstrated reasonable accuracy in identifying excessive inspiratory effort when values exceed 3.5 to 4.0 cm H_2_O and high precision in detecting low inspiratory effort at levels below 1.0 cm H_2_O. The accuracy of P0.1 readings across different ventilator models generally aligns well with reference method measurements, though precision may vary due to technical factors and patient-specific conditions like auto-PEEP. These findings underscore the utility of P0.1 as a practical tool for assessing respiratory drive and effort in mechanically ventilated patients, offering clinicians valuable insights for optimizing ventilation strategies and preventing P-SILI [37].

## 5. Management Strategies for Reducing P-SILI Risk

Since P-SILI is a concept that has been studied in animal models and small human studies, its true impact on the management of ARDS is currently unknown. Several studies have evaluated the effect of current ARDS management strategies on ameliorating the physiology of P-SILI. However, large clinical trials are needed to confirm these findings. Furthermore, ARDS is a heterogeneous disease, and designing clinical trials to assess the impact of P-SILI and targeted intervention requires better phenotyping and patient selection. In this section, we highlight relevant clinical literature on current ARDS management strategies and their impact on the physiology of P-SILI. We also highlight certain risk scores that can assist in the decision-making on when to transition from non-invasive to invasive mechanical ventilation.

### 5.1. Positive-End Expiratory Pressure (PEEP)

The effects of PEEP on P-SILI in ARDS have been extensively studied, with varying results [33,38,39].

#### 5.1.1. Impact on Lung Injury and Inflammation

Morias et al. investigated the effects of PEEP on lung injury in ARDS. Lower PEEP levels (5 cm H_2_O) were associated with increased inspiratory effort, higher lung stress in dependent regions, and more inflammation, while higher PEEP (15 cm H_2_O) levels mitigated these effects. Higher PEEP reduced inspiratory effort and tidal volume, recruiting atelectatic lung tissue and decreasing spontaneous breathing efforts through neuromechanical uncoupling [38]. Yoshida et al., in a porcine model of ARDS, demonstrated that optimizing PEEP can help mitigate the potentially harmful effects of spontaneous breathing in severe ARDS by reducing inspiratory effort, *pendelluft*, and tidal recruitment [33]. Pourfathi et al. demonstrated that PEEP appeared to contain pulmonary inflammation and injury progression by maintaining lung aeration, reducing anaerobic metabolism, limiting neutrophil recruitment/activation, and preserving lung mechanics in an experimental animal model of severe ARDS [40].

In contrast, some studies have also shown that PEEP did not affect *pendelluft*. Santini et al. showed that higher PEEP did not reduce or prevent *pendelluft* caused by higher inspiratory flows in ARDS patients [39]. The *pendelluft* appeared to be primarily flow-dependent rather than PEEP-dependent. Chi et al. found a weak negative correlation between PEEP and *pendelluft*, but only in specific subgroups of more severe patients. The overall effect across all patients was not significant [41]. These studies highlight the significant heterogeneity in lung injury and ARDS and the need for better phenotyping to understand which subgroup of patients may benefit from high-PEEP strategies in attenuating P-SILI.

#### 5.1.2. Effects on the Diaphragm

The use of PEEP in mechanical ventilation can have significant effects on the diaphragm. PEEP alters the diaphragm geometry by causing a caudal displacement of the diaphragm dome and shortening the zone of apposition, leading to reduced neuromechanical efficiency [42]. In the short term, high PEEP may attenuate patient efforts and reduce *pendelluft* and PL, thus reducing P-SILI. However, prolonged PEEP application can result in longitudinal muscle atrophy, which may cause additional diaphragm myotrauma when PEEP is withdrawn. Furthermore, PEEP can modulate respiratory drive and effort, potentially leading to diaphragm injury, and its level can influence diaphragm activity during expiration, contributing to myotrauma [42]. Therefore, setting optimal PEEP is crucial for both lung and diaphragm protective ventilation. In conclusion, while PEEP has shown promise in mitigating *pendelluft* and P-SILI, its effects are complex and not uniformly beneficial across all patients, thus highlighting the need for personalized approaches in mechanical ventilation strategies.

#### 5.1.3. PEEP in Special Populations

Guidelines for optimal PEEP settings in pediatric ARDS have not been standardized due to sparse studies in this special population. By consensus, pediatric intensivists prefer a low PEEP < 5 cm H_2_O with a range of 0–15 cm H_2_O and a high FiO_2_ strategy [43,44].

An optimal PEEP strategy can also be challenging in obese patients with ARDS. Although an observational study showed that higher PEEP is associated with better survival in obese ARDS patients, identifying optimal PEEP requires the use of tools such as esophageal manometry and P0.1 [45]. Electrical impedance tomography (EIT) has been utilized to identify heterogeneous ventilation and pendelluft in obese patients and can guide ventilator management, PEEP settings, and extubation strategies [46,47].

### 5.2. Awake Prone Positioning

Prone positioning is a recommended treatment approach in severe ARDS patients undergoing IMV. The PROSEVA trial demonstrated a significant reduction in 28-day and 90-day mortality in severe ARDS patients, leading to a significant change in how we manage mechanically ventilated ARDS patients. The physiologic benefits of proning are attributed to improved V/Q matching by the redistribution of blood and airflow, increased functional residual capacity, and a reduction in dead space ventilation. Hypothetically, similar effects could be anticipated in non-intubated patients. However, Grieco et al. studied the physiologic impact of awake-prone positioning in AHRF patients. They found that it increased the P/F ratio, reduced the respiratory rate, and promoted the distribution of tidal volume towards dependent regions of the lung with lower dynamic strain [48]. However, they also found that it increased end-expiratory lung impedance and pleural pressures (measured as esophageal pressures) without improving pendelluft, suggesting that it did not reduce the risk of P-SILI [48]. Alhazzani et al. did not find any significant reduction in the need for invasive mechanical ventilation (IMV) in COVID-19 patients undergoing awake proning [49]. Similarly, Nay et al. did not find any significant benefit in composite outcomes with awake-prone positioning in COVID-19 patients [50]. In contrast, Li et al., in a systematic review, identified that prone positioning reduced the risk of endotracheal intubation in COVID-19 patients requiring advanced non-invasive respiratory [51]. The utility of awake-prone positioning is still open for debate, and further studies are needed to identify its role in reducing the risk of P-SILI in non-intubated patients with ARDS.

### 5.3. Non-Invasive Respiratory Support: High-Flow Nasal Cannula (HFNC) and Non-Invasive Ventilation (NIV) Optimization

It is important to review common non-invasive respiratory support methods used in ARDS, which can curtail P-SILI and reduce the need for IMV. Two of the most common tools are the high-flow nasal cannula (HFNC) and non-invasive positive pressure ventilation (NIV/NIPV), which can provide PEEP and meet the patient’s respiratory flow demands.

**Figure 2:**
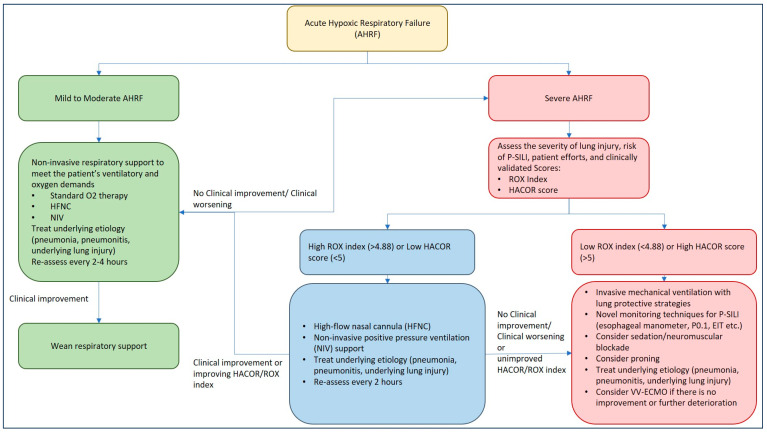
Management approach to acute hypoxic respiratory failure and P-SILI.

The evidence comparing HFNC and NIV for acute hypoxemic respiratory failure (AHRF) is mixed, with no clear superiority of one method over the other for most outcomes. While HFNC may offer advantages in patient comfort and potential mortality benefits in specific patient populations, NIV tends to provide greater oxygenation improvement with PEEP but carries risks of delayed intubation and potential lung injury from volutrauma and barotrauma, as delivered volumes often cannot be controlled if patient efforts are not curtailed. Several studies have compared NIV and HFNC to standard O_2_ therapy with conflicting results, which are highlighted in Table 1. The choice between HFNC and NIV for AHRF remains complex, with each method offering distinct advantages and potential drawbacks depending on the specific clinical scenario. Ultimately, the decision should be tailored to individual patient needs, considering factors such as comfort, respiratory efforts, oxygenation requirements, and the potential for complications, while maintaining vigilant monitoring for signs of respiratory deterioration that may necessitate escalation to invasive mechanical ventilation (Figure 2).

### 5.4. Monitoring Patients on Non-Invasive Support: ROX Index for HFNC Therapy and HACOR Score for NIV Therapy

It is imperative to closely monitor patients with ARDS on non-invasive respiratory support, as delay in recognizing treatment failure is associated with significant morbidity and mortality. As the goal of respiratory support is to provide lung rest and prevent further lung injury (P-SILI), dynamic respiratory monitoring using clinically validated tools may identify patients who may need IMV with lung protective strategies. Tonelli et al. evaluated 30 patients with acute hypoxic respiratory failure within 24 h of the NIV trial and monitored dynamic changes in PL and pleural pressures along with tidal volumes and inspiratory efforts [60]. Reduction in the magnitude of inspiratory efforts with NIV within the first 2 h of therapy was found to accurately predict treatment success, with a higher need for IMV in patients who continued to have higher inspiratory efforts [60]. The study highlighted the importance of monitoring respiratory mechanics with noninvasive respiratory support and the early identification of treatment failure. Depending on the method of noninvasive respiratory support, two commonly utilized tools, in addition to clinical judgment, include the ROX index for HFNC and the HACOR score for NIV.

#### 5.4.1. ROX Index for HFNC Therapy

The ROX (Respiratory rate-OXygenation) index was introduced in 2016 by Roca et al. as a novel prediction tool to identify the need for IMV in pneumonia patients with AHRF treated with HFNC [61]. It is calculated as [(SpO_2_/FiO_2_)/Respiratory Rate] and is typically assessed at 2, 6, and 12 h after HFNC initiation [61]. Since its introduction, the ROX index has been externally validated in various populations, including COVID-19 ARDS, making it a reliable bedside tool for clinical decision making [62,63,64,65]. Studies have shown that the ROX index demonstrates good diagnostic accuracy for predicting HFNC failure, with a summary receiver operator characteristic curve of 0.771 [66]. A meta-analysis suggests an optimal general cut-off of 5.23, which is higher than the originally proposed 4.88 [61,66]. The ROX index tends to perform best at 6–12 h after HFNC initiation, or later. By identifying patients at risk of P-SILI and guiding early IMV, the ROX index can help prevent unnecessary delays in escalating care, potentially reducing morbidity and mortality associated with P-SILI.

#### 5.4.2. HACOR Score for NIV Therapy

The HACOR (Heart rate, Acidosis, Consciousness, Oxygenation, and Respiratory rate) score is a valuable tool for predicting NIV failure in patients with AHRF. This scoring system evaluates five key physiological parameters that are crucial indicators of a patient’s respiratory status and development of P-SILI [67]. The HACOR score demonstrates good predictive power for NIV failure, with higher scores indicating a greater risk of failure and the potential need for IMV [67,68]. Using a score of >5 maintains a strong diagnostic accuracy in predicting NIV failure regardless of patient diagnosis, age group, or illness severity [67].

The HACOR score’s utility extends beyond prediction; it aids clinicians in making timely decisions regarding patient management, particularly in determining whether early intubation might be beneficial. Patients with high HACOR scores who undergo early intubation tend to have better outcomes, including lower hospital mortality rates, compared to those who receive delayed intubation [67,68]. The HACOR score has been clinically validated across various patient populations, including those with chronic obstructive pulmonary disease (COPD) and non-COPD patients with acute-on-chronic respiratory failure [69]. Its applicability has even been extended to patients receiving HFNC therapy. A HACOR score <6 after 1 h was associated with <85% risk of treatment failure [70]. An updated HACOR score was developed, which improved upon the original by incorporating six pre-NIV variables: pneumonia, cardiogenic pulmonary edema, pulmonary ARDS, immunosuppression, septic shock, and SOFA score. This enhancement significantly increased its predictive power in patients with hypoxemic respiratory failure and allows for classifying patients into low, moderate, high, and very high-risk categories for NIV failure [71].

### 5.5. Invasive Mechanical Ventilation (IMV) Strategies

#### 5.5.1. Lung-Protective Ventilation

Lung-protective ventilation has become a cornerstone in managing patients with ARDS, aiming to minimize VILI [72]. The landmark ARDSNet ARMA trial demonstrated a significant reduction in mortality when using lower tidal volumes of 6 mL/kg (predicted body weight) compared to traditional volumes of 12 mL/kg [73]. To prevent both VILI and P-SILI, clinicians should focus on using low tidal volumes, limiting plateau pressures to below 30 cm H_2_O, and carefully managing positive end-expiratory pressure (PEEP).

#### 5.5.2. Driving Pressure

Driving pressure (DP) has emerged as a crucial physiological parameter in understanding and mitigating lung injury in mechanically ventilated patients, as sicker lungs are stiffer lungs. Amato et al. demonstrated that high DP was a better predictor of mortality than high tidal volume in patients with ARDS [74]. Each 7 cm H_2_O increase in DP raised the risk of death by 41%, even with protective ventilation settings [74]. This finding underscored the importance of adjusting ventilation strategies based on DP to minimize VILI. The work of Yoshida et al. has established a clear correlation between transpulmonary pressure and P-SILI in severe ARDS [3,14,15,18,33]. These findings have important clinical implications, highlighting the need for the careful monitoring of spontaneous breathing efforts and PL/DP in vulnerable patients. The recognition that SB can contribute to lung injury has led to a broader understanding of mechanical ventilation as not just supportive but also potentially prophylactic in preventing further lung damage when tailored to reduce P-SILI [75].

#### 5.5.3. Sedation and Neuromuscular Blockade

Sedation plays a crucial role in implementing lung- and diaphragm-protective (LDP) ventilation strategies. Respiratory efforts can become excessive if SB is permitted in severe ARDS, potentially leading to P-SILI. Therefore, titrating sedation to attenuating respiratory efforts rather than traditional sedation scores may make logical sense as traditional scores correlate poorly to respiratory drive [76]. The goal is to achieve a balance that avoids both insufficient effort (leading to diaphragm disuse atrophy) and excessive effort [76]. In some cases, additional interventions like partial neuromuscular blockade (NMB) may be necessary to control excessive respiratory effort when sedation alone is insufficient.

Doorduin et al. explored a novel strategy to deliver LDP ventilation while maintaining diaphragm activity in patients with ARDS [77]. They found that partial NMB using low-dose rocuronium facilitated LDP ventilation by reducing tidal volumes and PL to safer levels while maintaining some diaphragm activity. However, this approach was associated with mild hemodynamic side effects and the development of hypercapnic acidosis [77]. NMB limits PL; this effect is also augmented by less lung de-recruitment during expiration, greater lung recruitment during inspiration, and overall decreased expiratory muscle activity [78]. Forel et al. demonstrated that NMB was associated with lower levels of pulmonary inflammatory markers within the first 48 h of IMV [79]. Another clinical trial of 56 patients across four medical intensive care units demonstrated that NMB in the first 48 h improved the P/F ratio [80].

The ACURASYS trial comprising 340 ARDS patients was the first landmark study to demonstrate a higher adjusted overall survival at 90 days in patients receiving NMB with deep sedation within the first 48 h of IMV [81]. A subgroup analysis of patients with a P/F ratio <120 had a higher probability of survival. Patients with NMB were also less likely to develop barotrauma consequent to VILI, which was most likely to occur within the first 48 h [81]. However, the ACURASYS trial was performed during a time when deep sedation practice was considered the norm for ARDS; this is no longer the practice [82]. In contrast, the ROSE trial, a multicenter, unblinded randomized control trial comprising 1008 patients with moderate-to-severe ARDS, did not observe any statistically significant reduction in 90-day mortality with NMB coupled with deep sedation versus light sedation alone in the control group [83]. Further stratification of the 90-day mortality percentage based on the P/F ratio (<, or >= 120), duration of ARDS prior to randomization, and hospital tercile for prior NMB use did not reveal any significant difference [83].

Perhaps the main reason for the difference in these results between the ACURASYS and ROSE trials is the difference in sedation levels in the control group. While deep sedation may abolish the classic “patient–ventilator dyssynchrony”, it may introduce a proclivity towards reverse triggering, a recently recognized form of dyssynchrony [84]. Reverse triggering can predispose VILI by increasing tidal volume and PL; NMB can blunt these effects. Thus, the higher VILI observed in the control group in ACURASYS as opposed to that in ROSE is likely due to deep sedation without NMB in the former trial [85,86].

### 5.6. Veno-Venous ECMO to Facilitate Lung Rest

Veno-venous extracorporeal membrane oxygenation (VV-ECMO) can significantly facilitate lung-protective ventilation strategies in patients with severe ARDS, failing IMV. A physiological trial by Dianti et al. found that patients on VV-ECMO were more likely to achieve LDP ventilation targets (Pes swing −3 to −8 cm H_2_O and ΔPLdyn ≤ 15 cm H_2_O) compared to those not on VV-ECMO [76]. Increasing sweep gas flow in VV-ECMO patients can effectively attenuate respiratory effort and lung-distending pressure. The use of VV-ECMO allowed for a better control of spontaneous breathing and respiratory effort, making it easier to implement protective ventilation strategies. A recent scoping review emphasized the role of VV-ECMO in preventing barotrauma by enabling ultra-protective ventilation, avoiding invasive ventilation, and extubating while on ECMO [87]. In a recent prospective observational study, clinical and gas exchange criteria based on an Extracorporeal Life Support Organization (ELSO) guideline with further clinical validation by a dedicated intensivist enabled safe extubation in patients with severe ARDS undergoing VV-ECMO, thus offering a standardized approach for the same [88]. “Awake ECMO” without invasive ventilation in patients with respiratory failure is a current key area of exploration; however, whether ARDS patients would benefit from awake ECMO is doubtful: a recent systematic review revealed an “awake ECMO” failure rate of close to 40% in ARDS sub-group [89]. Overall, VV-ECMO appears to be a valuable tool in managing severe respiratory failure while minimizing the risk of P-SILI and diaphragm dysfunction [76].

## 6. Knowledge Gaps and Future Directions

Despite significant growth in the medical literature pertaining to the understanding and management of ARDS and P-SILI, several knowledge gaps require further investigation. The impact of the gut microbiome (gut-lung axis), phenotyping of ARDS based on the inflammatory response, epigenetics, and environmental contribution to ARDS needs to be further investigated to allow for the identification of optimal management strategies. The concept of P-SILI is relatively novel and presents as a spectrum in lung injury depicting the impact of spontaneous breathing on lung injury. The current literature is hypothesis-generating and requires clinical validation in large-scale trials to identify the prognostic impact of P-SILI and management strategies to ameliorate it. Novel investigational tools need prospective validation to allow for evidence-based utilization in daily clinical practice.

## 7. Conclusions

With advances in the understanding of respiratory mechanics in ARDS, there is an evolving recognition of the differential impact of spontaneous breathing across the spectrum of the disease. P-SILI is a complex form of lung injury that occurs due to a patient’s respiratory efforts, leading to increased stress/strain, pendelluft, and microvascular injury, perpetuating the cycle of inflammation and lung damage. With novel investigational tools and techniques, such as esophageal manometry, electrical impedance tomography, static and dynamic assessment of a patient’s respiratory efforts, and diaphragmatic activity, P-SILI can be identified and attenuated. Using non-invasive respiratory support such as HFNC or NIV is essential to reduce the need for IMV. However, close clinical monitoring with the guidance of well-validated tools such as the ROX index or HACOR score can assist providers to consider IMV in key decision making. Lung protective mechanical ventilation and individualized care with optimal PEEP and judicious use of sedation and NMB remain the cornerstone of managing ARDS and P-SILI prevention. Further clinical studies are needed to assess treatment strategies and prognostic impact of P-SILI in patients with ARDS.

## Figures and Tables

**Table 1:** Clinical trials and key studies comparing NIV and HFNC compared to standard O_2_ therapy in AHRF patients.

Authors/Study	Patient population	Study Design	Sample Size	Key Findings	Limitations
FLORALI Study (Frat et al., 2015) [52]	AHRF patients	Multicenter open-label RCT	310 patients	Lower 90-day mortality in HFNC compared to standard O_2_ therapy. Lower intubation rates, ventilator-free days with HFNC in patients with P/F < 200	Lower EPAP in NIV cohort; Sicker patients in NIV cohort compared to HFNC cohort
HOT-ER Study (Jones et al., 2016) [53]	AHRF patients	Pragmatic open RCT	303 patients	HFNC did not reduce the need for IMV in the emergency department compared to standard O_2_	Small sample size (underpowered); Unblinded study
LUNG SAFE Study (Bellani et al., 2017) [54]	ARDS patients	Observational multicenter study	436 patients (non-invasively ventilated)	NIV use was associated with higher ICU mortality in patients with P/F < 150	Patient severity scores were not collected; HFNC not included; NIV treatment for days 1–2 included in the NIV arm (shorter duration excluded) Unblinded study
HENIVOT Trial (Grieco et al., 2021) [55]	Moderate-to-severe AHRF in COVID-19 patients	Multicenter RCT	109 patients	No difference in respiratory support-free days and mortality in NIV vs. HFNC groups. The rate of endotracheal intubation and IMV-free days lower in NIV compared to HFNC group	Small sample size (Underpowered); Awake prone positioning was not standardized
RECOVERY-RS Trial (Perkins et al., 2022) [56]	AHRF in COVID-19 patients	Parallel group, adaptive RCT	1273 patients	NIV significantly reduced the risk of endotracheal intubation compared to standard O_2_ therapy. No significant difference in HFNC vs. NIV	Did not achieve planned sample size (underpowered)
Pitre et al., 2023 [57]	AHRF	Systematic review and Network Meta-analysis	36 trials (7046 patients)	Helmet NIV reduces mortality, risk of IMV, and hospital/ICU stay duration. HFNC reduces the risk of IMV	Heterogenous population
Munroe et al., 2024 [58]	AHRF patients	Single-center propensity-matched retrospective study	1154 patients	NIV compared to HFNC, is associated with lower mortality, fewer hours of respiratory support, and fewer pulmonary adverse events	Single-center, retrospective study
RENOVATE Trial (Israel et al., 2024) [59]	AHRF patients divided into subgroups: non-immunocompromised, COPD, acute pulmonary edema, COVID-19	Non-inferiority, RCT Bayesian hierarchical model	1766 patients	HFNC non-inferior to NIV for time to endotracheal intubation or death in 7 days in all groups except immunocompromised cohort (low sample size)	Low EPAP setting in the NIV group; Smaller sample size in the immunocompromised cohort

## Abbreviations

acute hypoxic respiratory failure (AHRF); acute respiratory distress syndrome (ARDS); computed tomography (CT); diaphragm electrical activity (EAdi); driving pressure (DP); end-expiratory lung volume (EELV); esophageal pressure (Pes); electrical impedance tomography (EIT); high-flow nasal cannula (HFNC); inspiratory muscle pressure (Pmus); invasive mechanical ventilation (IMV); lung- and diaphragm-protective ventilation (LDP); neuromuscular blockade (NMB); non-invasive ventilation (NIV); pressure support ventilation (PSV); positive end-expiratory pressure (PEEP); partial pressure of oxygen to fractional inspiratory oxygen ratio (P/F ratio); patient-self inflicted lung injury (P-SILI); right ventricular (RV); spontaneous breathing (SB); transpulmonary pressure (PL); ventilator-induced lung injury (VILI); ventilation/perfusion (V/Q); veno-venous extracorporeal membrane oxygenation (VV-ECMO).

## Appendix A

**Table A1:** Inclusion and exclusion criteria for the literature review.

Criteria	Inclusion	Exclusion
Publication Date	2000–2025	Before 2000
Study Type	Peer-reviewed clinical studies, systematic reviews, and meta-analyses	Abstract only, proposals, or studies without final results
Language	English	Non-English
Patient Population	All genders, adult populations	Studies focused solely on pediatric populations (except in section highlighting special population)
Data Sources	All (facilities, community-based, national, sub-national databases)	Studies with incomplete or unavailable full text
Relevance	Studies that mention outcomes of interest	Studies that do not report relevant outcomes

**Table A2:** Level of evidence for key management recommendations.

	Management Strategy	Supporting Studies	Level of Evidence
1	High PEEP to reduce P-SILI in severe ALI	RCTs, meta-analyses	Level I
2	Awake Prone Positioning	RCT, systematic review	Level I
3	Non-invasive ventilation (NIV) vs. High-Flow Nasal Cannula (HFNC)	Systematic reviews, mixed RCTs	Level II
4	Use of neuromuscular blockade in early ARDS	Large RCTs	Level I
5	VV-ECMO for lung rest in refractory hypoxemia	Systematic review, scoping review, observational studies.	Level III
